# Development of a Gold Nanoparticle-Based Sensor for Authentication of Organic Milk Based on Differential Levels of miRNA

**DOI:** 10.3390/nano14161364

**Published:** 2024-08-19

**Authors:** Karelmar Lopez-Benitez, Patricia Alcazar-Gonzalez, Loubna Abou el qassim, Mª Teresa Fernandez-Argüelles, Fernando Vicente, Luis J. Royo, Mario Menendez-Miranda

**Affiliations:** 1 Regional Service for Agrofood Research and Development (SERIDA), 33300 Asturias, Spain; karelmar@serida.org (K.L.-B.); loubna@serida.org (L.A.e.q.); fvicente@serida.org (F.V.); royoluis@uniovi.es (L.J.R.); 2 Department of Physical and Analytical Chemistry, Faculty of Chemistry, University of Oviedo, 33006 Asturias, Spain; alcazarpatricia@uniovi.es (P.A.-G.); fernandezteresa@uniovi.es (M.T.F.-A.); 3 Department of Functional Biology, Genetics, University of Oviedo, 33006 Oviedo, Spain

**Keywords:** dairy production, visual bioassay, genetic detection, gold nanosensor, miRNA detection, organic milk, biomarkers

## Abstract

Dairy production systems significantly impact environmental sustainability, animal welfare, and human health. Intensive farming maximizes output through high-input practices, raising concerns about environmental degradation, animal welfare, and health risks from antibiotic residues. Conversely, organic farming emphasizes sustainable practices, animal welfare, and minimal synthetic inputs, potentially enhancing biodiversity, soil health, and milk quality. MicroRNAs (miRNAs), non-coding RNAs regulating gene expression, are promising biomarkers due to their response to various conditions. In this study, miRNAs bta-miR-103 and bta-miR-155, which are abundant in milk from pasture-fed cows, were selected. Additionally, bta-miR-215, which is abundant in milk fat from intensive systems, was also studied, in order to differentiate dairy production systems. A novel, cost-effective gold nanoparticle (AuNP)-based sensor was developed for miRNA detection, leveraging the unique plasmonic properties of AuNPs for visual detection. The method involves functionalizing AuNPs with complementary RNA probes and detecting miRNA-induced aggregation through colorimetric changes. This rapid, results in 30 min, and sensitive, visual limit of detection of 200 nM, assay requires minimal instrumentation and can be easily interpreted, offering significant advantages for field implementation in characterizing dairy production systems. This study demonstrates the successful application of this sensor in detecting miRNAs in 350 nM miRNA spiked raw milk, highlighting its potential for in situ dairy industry applications.

## 1. Introduction

The dairy industry is a critical component of global agriculture, providing essential nutrients to billions of people around the world. However, the methods of dairy production have significant implications for environmental sustainability, animal welfare, and human health [1]. The two predominant systems of dairy farming—extensive and intensive—represent contrasting approaches to these issues [2]. Within extensive systems, organic production is a special type of livestock farming with specific and concrete regulations.

Intensive dairy production focuses on maximizing output through the use of high-input practices [3], including concentrated feed, advanced breeding techniques, and frequent use of pharmaceuticals [4]. While this method significantly increases milk yield and can meet high consumer demand efficiently [5], it raises concerns about environmental degradation, animal welfare issues, and potential health risks from antibiotic residues and higher greenhouse gas emissions.

In contrast, organic dairy production emphasizes sustainable farming practices, animal welfare, and minimal use of synthetic inputs. In the European Union (EU), the legislation governing organic farming is the Council Regulation 2092/91 on Organic Agricultural Production. This law has undergone modifications such as the case of Regulation 1804/1999, which incorporates animal production, and Regulation (EU) 2018/848, which establishes the standards for the production, processing, marketing, labeling, and control of food as well as the importation of organic food (European Commission, 2018). This system relies on organic feed, access to pasture, and a prohibition of routine antibiotics and hormones. Advocates argue that organic dairy farming can enhance biodiversity, improve soil health, and produce milk with potential health benefits due to higher concentrations of beneficial fatty acids and antioxidants [6,7,8].

MicroRNAs (miRNAs), non-coding RNAs of 21–25 nucleotides that regulate gene expression post-transcriptionally by binding to specific mRNA targets, are promising biomarkers across various fields due to their ability to vary in response to disease, diet, stress, physiological states, and other factors. The presence, absence, or differential levels of specific miRNAs can indicate particular conditions, demonstrating their specificity and ability to differentiate between specific states [9,10,11,12,13]. Using miRNAs from body fluids such as milk offers the advantages of easy, non-invasive sampling and low cost. Moreover, miRNAs in milk are highly resistant to acidic pH and RNase enzymes, making them stable and robust against industrial processing [14,15,16]. Milk, in particular, is a rich source of miRNAs [17] and their expression is influenced by the genetic context [18] and environmental factors [19,20,21]. Changes in diet [22], exercise [23], and stress [24] have all been shown to affect gene expression in the mammary gland.

In this work, three different miRNAs have been selected, specifically bta-miR-215, bta-miR-155, and bta-miR-103. Bta-miR-215 is significantly more abundant in milk fat produced in intensive systems than in extensive systems [2]. Moreover, this biomarker has significantly lower levels in organic herds [25]. Regarding bta-miR-155, it has been found that its levels were higher in milk fat from cows raised on pasture farms than in cows raised on intensive farms and fed mainly corn silage. Referring to bta-miR-103, the milk fat levels of bta-miR-103 were higher on farms that included fresh grass in the diet, either grazed or mown and offered at the manger [26]. In addition, in trials carried out under controlled management and feeding conditions, the bta-miR-155 and bta-miR-103 markers also showed higher levels in milk from cows fed on pasture than the same cows when kept in stalls [27].

Nowadays, there are numerous tools available for miRNA detection, such as Northern blot [28], quantitative real-time polymerase chain reaction (qRT-PCR) [29], sequencing [30], cloning [31], and in situ hybridization [32]. However, some of these methods have certain limitations, as they are often time-consuming, require large sample volumes, and consist of multiple experimental steps [33,34]. Additionally, in some cases, the sensitivity is not good enough for detecting miRNA at relevant concentration levels, as the ideal scenario would be to detect them at subclinical levels [35]. Methods based on hybridization, such as sequencing, cloning, and in situ hybridization, and methods based on amplification, such as qRT-PCR, provide high specificity and sensitivity for the detection of genetic targets [36,37]. However, they require a delicate and labor-intensive primer design due to the short length of miRNAs, as well as complex sample preparation involving multiple experimental steps, including miRNA modification, which requires highly qualified personnel to perform both the experimental work and subsequent data analysis, resulting in higher costs [38].

In this sense, recent advances in nanotechnology have introduced novel approaches to nucleic acid detection, with gold nanoparticle (AuNP) sensors emerging as a promising solution [39,40]. Au NPs possess unique optical, electronic, and chemical properties that make them ideal for biosensing applications [40,41,42]. Their high surface area-to-volume ratio, ease of functionalization with biomolecules, and ability to facilitate signal amplification contribute to their effectiveness in detecting low concentrations of nucleic acids.

Colorimetric methodologies using AuNPs offer a convenient option for rapid and cost-effective miRNA detection. Although a few colorimetric assays using AuNPs for miRNA detection have been described, most require lengthy analysis times and amplification steps involving enzymes [43,44]. Ren, M. et al. [45] reported a highly sensitive miRNA detection method based on AuNP aggregation followed by resonance light scattering measurements. However, this approach requires complex and expensive instrumentation, limiting its use for routine analysis.

Building on these techniques, this study presents the design, optimization, and evaluation of an assay for miRNA detection using visual optical changes. This method leverages the surface plasmon resonance (SPR) absorption changes that occur when AuNPs aggregate in the presence of the target molecule [46,47,48]. To ensure selectivity, AuNPs are modified with short RNA sequences complementary to the target miRNA sequence. In addition to measuring the absorbance spectrum using a UV–vis spectrophotometer, the samples are spotted onto a C18 silica thin-layer chromatography (TLC) plate, which retains the color of the solution mixture and prevents dissociation of the formed aggregates, providing a visual detection. This strategy offers a rapid, cost-effective, and highly sensitive method for miRNA detection. It avoids the need for sophisticated templates, and the readout can be performed visually without requiring complex and expensive instrumentation [46,47].

This paper presents the development and application of a gold nanoparticle-based sensor for the rapid and accurate detection of miRNA nucleic acids in dairy products for the differentiation of the production system. The sensor leverages the unique plasmonic properties of gold nanoparticles, enabling visual detection through colorimetric changes in the presence of the analyte, in order to identify the dairy production-based system.

## 2. Materials and Methods

### 2.1. Materials and Reagents

All reagents were of analytical grade and used without any further purification unless otherwise mentioned. The thiolated RNA strands employed to functionalize the AuNPs surface can be found in Table 1 and were obtained from Metabion (Panegg, Germany). Hydrogen Tetrachloroaurate Trihydrate (HAuCl_4_ ∙ 3H_2_O) and Hydrochloric Acid 1N were purchased from Sigma-Aldrich (St. Louis, MO, USA). Tri-sodium Citrate dihydrate (Na_3_C_6_H_5_O_7_ ∙ 2H_2_O), magnesium chloride hexahydrate (MgCl_2_ ∙ 6H_2_O), TBE (5X), Tris + borate + EDTA, Tween^®^20 (C_58_H_114_O_26_), and Potassium Chloride (KCl) were purchased from Thermo Fisher Scientific (Waltham, MA, USA). Tris hydrochloride (C_4_H_11_NO_3_ · HCl) was purchased from Boehringer Ingelheim International GmbH (Ingelheim am Rhein, Germany). Potassium Hydroxide pellets were purchased from BDH Laboratory Supplies (Dubai, United Arab Emirates). Thiolated polyethylene glycol (mPEG-SH_1000_) was purchased from Laysan Bio, Inc. (Arab, AL, USA). Agarose D1 Low EEO was purchased from Condalab (Madrid, Spain). TLC Aluminum Sheets Silica gel 60 F254 was purchased from Merck KGaA (Darmstadt, Germany). Milk samples were obtained from commercial farms located in Asturias, a region on the northern coast of Spain. Asturias is a region with a great livestock tradition and one of the main production areas of milk in Spain. miRNA extraction was performed using QIAzol lysis reagent from QIAGEN (Hilden, Germany) and mirVana microRNA isolation kit from Thermo Fisher Scientific (Waltham, MA, USA).

### 2.2. Synthesis of Gold Nanoparticles

**Figure 1:**
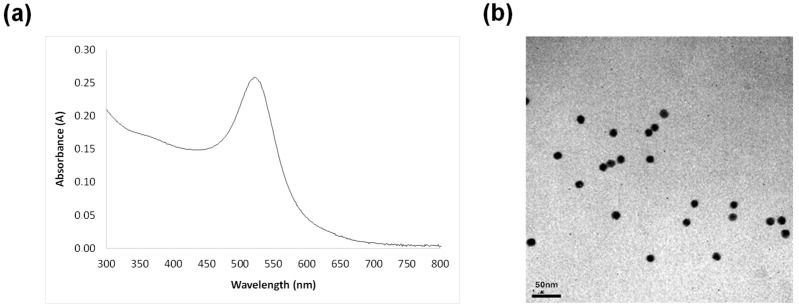
(**a**) Absorbance spectrum of the synthesized AuNPs; (**b**) TEM image of AuNPs.

Gold nanoparticles (AuNPs) were synthesized according to a method detailed in a prior publication [47]. Size, shape, and size distribution of the obtained AuNPs were evaluated using dynamic light scattering (DLS) and Transmission Electron Microscopy (TEM). As shown in Figure 1, the mean diameter of the produced AuNPs was determined to be 16 ± 1 nm (n = 100), with a PDI of 0.12 ± 0.04 and a zeta potential of −21 mV in aqueous solution containing 1 mM KCl at pH 7. This negative value suggests that electrostatic repulsion may help in the stabilization of the suspensions.

### 2.3. Functionalization of AuNPs

**Figure 2:**
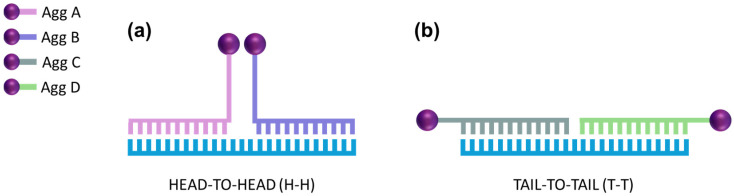
(**a**) Scheme of the Head-to-Head (H-H) conformation for the hybridization process. (**b**) Scheme representation of the Tail-to-Tail (T-T) conformation for the hybridization process.

Agg/A and Agg/B were designed to hybridize in a Head-to-Head (H-H) conformation with their specific analyte, while Agg/C and Agg/D were engineered to produce a Tail-to-Tail (T-T) conformation once hybridized, as shown in Figure 2.

AuNPs were functionalized with each probe summarized in Table 1 (Agg/A/B/C/D) for their respective analytes (bta-miR-103/155/215). In this sense, it is worth mentioning that AggAs and AggCs probes present a 5′-thiol modification while AggBs and AggDs present a 3′-thiol modification. Each of these sequences are partially complementary to one of the target analytes bta-miR-103, bta-miR-155, and bta-miR-215, respectively, and have a poly-A chain of nucleotides between the thiol modification and the complementary sequence, acting as a spacer to facilitate their binding to the analytes.

For the functionalization process, the bioconjugation took place by mixing 20 μL of AuNPs (100 nM) with 20 µL of each Agg probe at different concentrations in binding buffer (ultrapure water with 0.01% *v/v* Tween-20 and 90 mM Tri-sodium Citrate HCl buffer, pH = 3) for 30 min at room temperature. Afterwards, in order to improve the colloidal stability of the NPs, thiolated polyethylene glycol (mPEG-SH_1000_) was added to block any DNA-free areas that could be present on the AuNPs’ surface. For this purpose, each set of surface-modified AuNPs was mixed with 10 μL of mPEG-SH1000 (2 mM), and the mixture was incubated at 60 °C for 30 min. Then, a purification step to remove the excess mPEG-SH1000 from the supernatant was carried out by three centrifugation cycles (16,000× *g*) at 4 °C for 30 min. Finally, a sample dilution with ultrapure water with 0.01% *v/v* Tween-20 was carried out to obtain a final concentration of 10 nM AuNPs, based on an extinction coefficient of 4.92 × 10^8^ M^−1^ cm^−1^ at λ = 517 nm for 16 nm AuNPs.

### 2.4. Method Development

For the development of the analytical methodology, different parameters were evaluated and optimized. Specifically, the concentration of magnesium chloride, the hybridization temperature, and the incubation time were optimized [49,50,51,52]. Additionally, two assay formats were tested, modifying the conformation of the aggregation probes with the analyte after hybridization [53].

**Figure 3:**
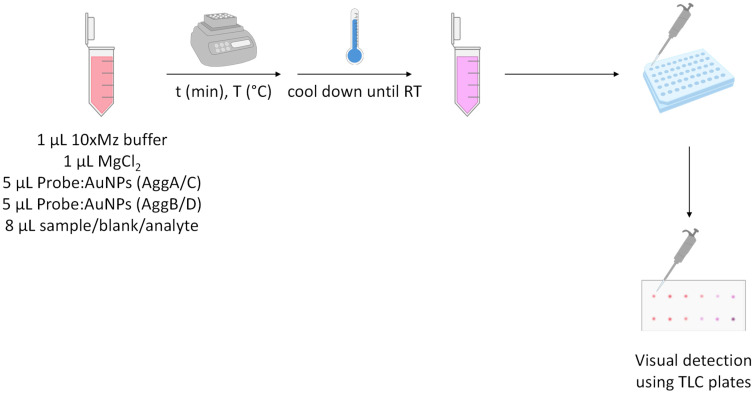
Scheme of the detection assay.

For the detection assay, see Figure 3. Briefly, 2 μL of the solution (being the blank, a known concentration of the analyte, or the sample with unknown concentration of the analyte) was mixed with 5 μL of AuNPs functionalized with AggA/C and 5 μL of AuNPs functionalized with AggB/D, depending on whether the desired conformation is H-H or T-T. Both bioconjugates were employed at a concentration of 10 nM, in 8 μL of a buffer solution containing MgCl_2_∙6H_2_O and 10xMz (0.1 M Tris-HCl and 0.5 M KCl, pH = 8.3). The mixture was incubated to allow hybridization of the functionalized AuNPs with the analyte. Once the reaction was completed, 2 μL of the reaction mixture was spotted onto a TLC plate to observe the color difference between aggregated and non-aggregated AuNPs. The remaining reaction mixture was diluted with ultrapure water to measure the shift in the maximum wavelength of the SPR peak using UV–Vis spectroscopy.

### 2.5. Sample Pre-Treatment

The milk samples, 6 obtained from commercial farms, were pre-treated with QIAzol lysis reagent from QIAGEN (Hilden, Germany) and mirVana microRNA isolation kit from Thermo Fisher Scientific (Waltham, MA, USA) following the instructions of the manufacturer.

Tubes containing 45 mL of each milk sample were centrifuged at 1500× *g* for 20 min. The fat layer was transferred to fresh 15 mL RNase-free tubes, and then QIAzol lysis reagent was added (1 mL per milk fat gram). Tubes were vortexed until the fat was thoroughly dispersed, and samples were stored at −80 °C until miRNA extraction. Finally, microRNA was extracted from 2 mL of milk fat with QIAzol using the mirVana miRNA isolation kit.

The first step of the protocol for the miRNA extraction involves an organic extraction that begins with the addition of 4 mL of lysis/binding buffer to 2 mL of the lysate (fat + QIAzol). Then, 200 µL of miRNA Homogenate Additiveµ was added, and after mixing well, the mixture was incubated on ice for 10 min. Next, 6 mL of Acid Phenol: Chloroform was added and mixed. The mixture was centrifuged for 5 min at a maximum speed of 10,000× *g* at room temperature to separate the aqueous and organic phases. The supernatant was transferred to a new 15 mL tube without disturbing the interphase.

The second and final step is the enrichment of small RNA. For this step, one-third volume of 100% ethanol is added to the recovered volume. The lysate/ethanol mixture was passed through filter cartridges by centrifugation. After filtering the total recovered volume for each sample, the filtrate was collected in 15 mL tubes. Then, two-thirds volume of 100% ethanol was added to the filtrate. For each sample, the total volume was passed again through new filter cartridges; in this case, the flow-through was discarded, and the filter was washed three times with the provided washing solutions. Finally, the column was transferred to new tubes, and 100 µL of nuclease-free water preheated to 95 °C was added to elute the small RNA from the column by centrifugation for 1 min. The collected small RNA was stored at −80 °C.

## 3. Results and Discussion

### 3.1. Method Development

#### 3.1.1. Assay Optimization

In order to optimize the assay conditions, 500 nM analyte was used, and when optimizing one condition, the rest were kept constant. In this sense, different parameters have been evaluated, the probe to AuNPs ratio (Aggs:AuNPs) for the superficial modification of the nanoparticles, the MgCl_2_ concentration, hybridization time, and temperature for both probe configurations H-H and T-T.

##### Agg Probe-AuNP Ratio Optimization

In order to achieve the maximum possible surface density of thiolated DNA strands, different molar ratios of DNA were studied. The bioconjugation procedure previously described was carried out, modifying the concentrations of added thiolated DNA while keeping the concentration of AuNPs constant. Subsequently, a 1% agarose gel electrophoresis was performed to determine the necessary amount of each DNA probe needed to saturate the surface of the AuNPs. 

There is a trade-off between two main factors in the migration of AuNPs. On the one hand, as the DNA ratio increases, the negative charge of the surface coating, provided by the DNA strands, also increases, which promotes a higher migration speed. On the other hand, the size of the bioconjugate also increases due to a higher amount of DNA on the AuNP surface, which slows down its movement along the agarose gel. The AuNP surface is considered saturated with Agg strands when enough DNA has been added so that the bands migrate the same distance in the gel. Thus, additional DNA would not result in greater surface coverage of the AuNPs, making it unnecessary to use more DNA since it would not achieve further coverage. Therefore, the optimal molar ratios are selected based on the point at which the bands migrate at a constant distance.

**Figure 4:**
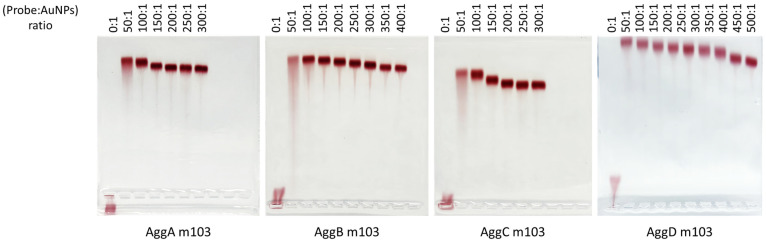
Agarose gel electrophoresis was carried out to optimize the concentration of AggA, AggB, AggC, and AggD for the detection of bta-miR-103. Bioconjugation with different molar ratios of each DNA probe per functionalized AuNP is shown.

Figure 4 shows an image of the bioconjugates after performing gel electrophoresis for the bta-miR-103 as an example, where it can be observed that, from a certain molar ratio of DNA to AuNPs, the migration distance of the bands remains constant.

The same procedure was carried out for the rest of the probes. As can be seen in Figure 4, the molar ratios selected for AggA/B/C/D for bta-miR-103 were 250:1, 350:1, 250:1, and 500:1, respectively. The results obtained for the rest of the Agg probes are summarized in Table 2.

The differences in molar ratios between probes, as well as the increase in bandwidth at low molar ratios for bioconjugates, can be explained by the structural differences between the probes. The molecules used to modify the ends of the DNA with a thiol functional group differ in structure depending on whether the modification is at the 5′ or 3′ end. Since these strands are antiparallel to each other, the thiol groups are located at opposite ends to allow each strand to hybridize with its corresponding analyte. Specifically, probes AggA and AggC are thiolated at the 5′ end, and probes AggB and AggD are thiolated at the 3′ end. This latter modification is chemically more complex and labile, contributing to greater instability of the bioconjugate-containing probes AggB and AggD compared to probes AggA and AggC, as well as requiring a higher amount of ligand to cover the AuNP surface, which in this case is true for all the assayed probes except for the AggB for bta-miR-215, which requires slightly less molar ratio than their 5′ thiolated counterparts.

##### MgCl_2_ Concentration, Hybridization Temperature, and Time Optimization

The effect of the different concentrations of MgCl_2_ on the detection of the analytes was evaluated as an increase in the number of cations present in the assay media reduces the repulsion effects between oligonucleotide chains (negatively charged), thereby positively affecting the hybridization process of the miRNAs [47,48,49,50]. However, the presence of salts in the medium can negatively impact the colloidal stability of AuNPs, as the repulsion between charged AuNPs is reduced, leading to their aggregation and precipitation. Since the work proposed here is based on the aggregation of AuNPs as a consequence of the presence of a certain miRNA, the addition of salts to the assay medium can result in the aggregation of AuNPs in the absence of the target sequence, leading to a false positive result. Therefore, to achieve a compromise, it is necessary to study the addition of salts to the assay medium to find the optimal concentration of cations that allows efficient hybridization of DNA strands without causing the aggregation of AuNPs in the absence of the analytes. In the present work, MgCl_2_ has been used as a salt to provide Mg^2+^ as a divalent cation to the medium.

**Figure 5:**
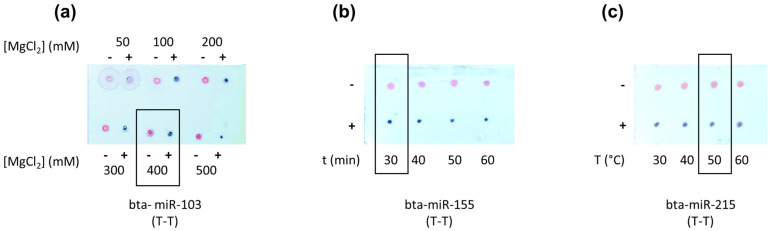
(**a**) Magnesium chloride optimization for the T-T assay format detection of bta-miR-103; (**b**) hybridization time optimization for the T-T assay format detection of bta-miR-155; (**c**) hybridization temperature optimization for the T-T assay format detection of bta-miR-215; − no presence of the analyte; + in presence of the analyte. TLC spots were carried out in triplicate (n = 3).

The MgCl_2_ concentration was evaluated from 0 to 500 mM, and Figure 5a shows as an example the MgCl_2_ optimization for the T-T format for the detection of the bta-miR-103. As can be seen, while at 50 mM of MgCl_2_ there is almost no aggregation, this aggregation can be seen from 100 mM. In this sense, 400 mM was selected, as it allows the detection of the analyte while not affecting the stability of the nanoparticles. The same procedure was performed for the rest of the probes; in all cases, the [MgCl_2_] = 400 mM was the selected concentration for further experiments.

The hybridization temperature and duration are crucial parameters to consider, as they directly depend on the concentrations of the sequences, their length, the G and C base content, and the concentrations of Na^+^ and Mg^2+^ cations present in the medium. All these parameters influence the so-called melting temperature (Tm) of DNA/RNA, which is the temperature at which at least 50% of the DNA/RNA strands are separated, i.e., denatured. In the hybridization process between two DNA/RNA sequences, it is necessary to reach this melting temperature for efficient hybridization to occur between complementary sequences.

The hybridization time was evaluated from 20 to 60 min and the temperature from 30 to 60 °C. Figure 5b,c show the results obtained for the T-T format assay detection for the bta-miR-155 and the bta-miR-215, respectively. As can be seen, all the conditions assayed resulted in positive results. However, 50 °C and 30 min were chosen as it is the melting temperature indicated by the manufacturer for the probes and the analytes (ranging from 44 to 54 °C) in order to ensure the effectiveness of the hybridization process.

#### 3.1.2. Analytical Characterization of the Method

The detection of analytes is carried out once all optimizations related to the experimental parameters affecting the method have been completed. For this, the H-H and T-T bioconjugated AuNPs are mixed with the assay buffer solution and the corresponding analyte standards at different concentrations, in order to determine the visual limit of detection (visual LOD) for the different analytes studied, as previously described. After 30 min of incubation at 50 °C, 2 μL of the mixture is placed on a TLC plate to observe the color difference between the aggregated and non-aggregated AuNPs. The remaining dispersion is diluted with ultrapure water to measure the color change resulting from the shift in the wavelength of the SPR peak maximum due to the aggregation of the AuNPs in the presence of the analyte.

**Figure 6:**
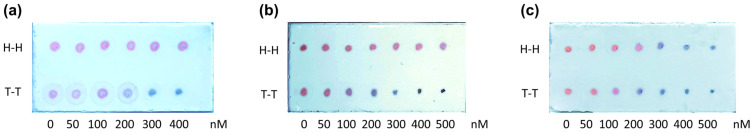
Visual limit of detection determination. (**a**) Visual LOD for bta-miR-103 using H-H (upper row) and T-T (lower row) assay formats. (**b**) Visual LOD for bta-miR-155 using H-H (upper row) and T-T (lower row) assay formats. (**c**) Visual LOD for bta-miR-215 using H-H (upper row) and T-T (lower row) assay formats. TLC spots were carried out in triplicate (n = 3).

As shown in Figure 6, the presence of the analyte at concentrations of 200 nM or higher results in a noticeable aggregation of the 15 nm AuNPs, changing the pink color to purple spots due to a shift in the SPR peak towards wavelengths of up to 555 nm for T-T conformations for the detection of bta-miR-103 and bta-miR-215. In this context, only a few methods have been reported for detecting microRNA based on the aggregation of AuNPs. Most of these methods involve complex amplification steps, which achieve lower detection limits but require temperature cycling, longer assay times, and spectrophotometric detection [43,44,54]. Additionally, some studies without amplification strategies have been reported [45,55]. However, these methods also require instrumental readouts using dynamic light scattering and ratiometric spectrophotometry, respectively, and longer assay times.

Moreover, the visual detection limit using the H-H conformation is higher for all three analytes, being 300 nM for bta-miR-103 and bta-miR-215. Interestingly, this higher LOD is more significant for the bta-miR-155 when using the H-H conformation, being 500 nM and 100 nM when using the H-H and the T-T conformation, respectively.

### 3.2. Application of the Developed Sensor

For the application and selectivity characterization of the developed methodology, four samples from commercial farms were selected. After the extraction of the total miRNA of the samples, and analyzing them via RT-PCR (see Table 3), the analytes were detected with over 25 cycle threshold (Ct) values. Additionally, bta-miR-30a-5p was also analyzed via RT-PCR as a control, as it is typically present in these types of samples [2], thus ensuring the successful miRNA extraction from the samples.

**Figure 7:**
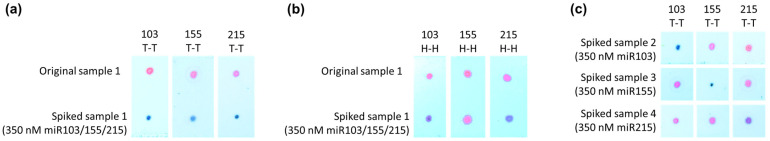
(**a**) Sample analysis using T-T conformations for raw milk (upper row) and spiked sample with 350 nM miR103, miR155, and miR215 (lower row). (**b**) Sample analysis using H-H conformations for raw milk (upper row) and spiked sample with 350 nM miR103, miR155, and miR215 (lower row). (**c**) Spiked sample 2 with 350 nM miR103 (upper row); spiked sample 3 with 350 nM miR155 (middle row); and spiked sample 4 with 350 nM miR215 (lower row). TLC spots were carried out in triplicate (n = 3).

Then, after performing the assay, it can be seen (see Figure 7a) that there is no aggregation when analyzing sample 1 as it is. However, after the addition of the corresponding analytes prior to the extraction of the miRNAs at a concentration of 350 nM, an aggregation due to the presence of the analytes can clearly be seen, confirming the selectivity of the methodology. As expected, the Ct values for all the spiked samples were lower, thus indicating a higher miRNA concentration than the original ones.

It is also worth mentioning that, as in the in-lab characterization, the detection process happens at a lower concentration when using the T-T conformation assay in comparison with the H-H format (see Figure 7a,b); this finding is in agreement with previous works [52].

In this context, when adding just one analyte to the samples, bta-miR-103, bta-miR-155, and bta-miR-215 were added to samples 2, 3, and 4, respectively, it can be seen (see Figure 7c) how their corresponding detection probes produce the aggregation, but no aggregation is found when using the detection probes of the other analytes, thus confirming that the aggregation of the AuNPs occurs due to the presence of the corresponding analyte and not just due to the presence of any miRNA as previously shown.

Regarding the visual LOD, when applying the developed methodology to milk samples, assuming a 70% miRNA extraction efficiency, the aggregation/detection of the analyte occurs at a concentration of 200 nM, which is consistent with the visual LOD obtained during the optimization of the method. Therefore, in view of the results obtained when applying the methodology to raw cow’s milk samples, it can be considered that this method can be successfully applied to the detection of bta-miR-103, bta-miR-155, and bta-miR-215.

## 4. Conclusions

A simple and cost-effective methodology has been developed for the rapid and sensitive detection of bta-miR-103, bta-miR-155, and bta-miR-215 based on the visualization of the color change in a colloidal AuNP dispersion, induced by their aggregation after incubation with the respective analytes. This analytical method is designed for the detection of the aforementioned miRNAs, recognized as biomarkers in the milk production system, by obtaining a visual detection limit of 200 nM without the need for any prior amplification step. Additionally, it has been demonstrated that different assay formats influence the analytical characteristics of the developed methodology. In fact, the way in which recognition probes are synthesized and their bioconjugation with nanoparticles takes place to generate different assay formats, i.e., H-H or T-T formats, has a direct influence on the visual aggregation. The results have shown that a higher sensitivity is achieved when the target miRNA recognition is performed using a T-T conformation.

The high selectivity of the assay, with no aggregation when analyzing raw cow’s milk samples that contain miRNAs with analytes present with Ct values over 25, can be ensured through the appropriate functionalization of the AuNPs with complementary oligonucleotides. It is noteworthy that the assay is completed in just 30 min under mild isothermal conditions (50 °C). Moreover, it is possible to perform the visual detection of analytes without the need for instrumentation, and the reading can be easily interpreted by non-qualified personnel, as it only requires observing a color change. Both aspects are significant advantages for the potential implementation of the method in the field. However, the step of extracting miRNAs from raw milk must be performed before measurement. Therefore, this presents a critical factor to be addressed for the potential development of a sensor and its implementation in the characterization of the milk production system directly on the farm as it also compromises the overall cost of the developed methodology.

In summary, this method developed for miRNA detection is rapid, low cost, and highly sensitive, and has been successfully applied to the detection of bta-miR-103, bta-miR-155, and bta-miR-215 in fat from raw milk samples, which are important features for the future development of rapid and in situ methods aimed at characterizing the dairy production system.

## Figures and Tables

**Table 1:** Probes used for the assay and conformation obtained after hybridization with the analyte. H-H meaning Head-to-Head conformation T-T meaning Tail-to-Tail conformation.

Conformation	Oligo Name	Sequence (5′→3′)
-	bta-miR-103	AGC AGC AUU GUA CAG GGG UAU GA
H-H	Agg/A m103	Thiol-C6-AAA AAA AAA AAA CAA TGC TGC T
	Agg/B m103	TCA TAG CCC TGT AAA AAA AAA-C3-Thiol
T-T	Agg/C m103	Thiol-C6-AAA AAA AAA ATC ATA GCC CTG
	Agg/D m103	TAC AAT GCT GCT AAA AAA AAA AA-C3-Thiol
-	bta-miR-155	UUA AUG CUA AUC GUG AUA GGG GU
H-H	Agg/A m155	Thiol-C6-AAA AAA AAA AGA TTA GCA TTA A
	Agg/B m155	ACC CCT ATC ACA AAA AAA AAA-C3-Thiol
T-T	Agg/C m155	Thiol-C6-AAA AAA AAA ACC CCT ATC ACG
	Agg/D m155	ATT AGC ATT AAA AAA AAA AAA-C3-Thiol
-	bta-miR-215	AUG ACC UAU GAA UUG ACA GAC A
H-H	Agg/A m215	Thiol-C6-AAA AAA AAA ATC ATA GGT CAT
	Agg/B m215	TGT CTG TCA ATA AAA AAA AAA-C3-Thiol
T-T	Agg/C m215	Thiol-C6-AAA AAA AAA ATG TCT GTC AAT
	Agg/D m215	TCA TAG GTC ATA AAA AAA AAA A-C3-Thiol

**Table 2:** Results obtained for the DNA:AuNP ratio optimization.

Oligo Name	(Probe:AuNPs) Ratio
Agg/A m103	250:1
Agg/B m103	350:1
Agg/C m103	250:1
Agg/D m103	500:1
Agg/A m155	300:1
Agg/B m155	350:1
Agg/C m155	350:1
Agg/D m155	400:1
Agg/A m215	400:1
Agg/B m215	350:1
Agg/C m215	400:1
Agg/D m215	400:1

**Table 3:** Cts obtained after RT-PCR analysis of the samples.

miRNA	Sample 1 (Ct)		Sample 2 (Ct)		Sample 3 (Ct)		Sample 4 (Ct)	
	Original	Spiked	Original	Spiked	Original	Spiked	Original	Spiked
103	33.7	22	28.7	27.5	24.5	16	27.3	27.5
155	29.1	22	20.1	21.2	27.3	28.5	25.7	18.5
215	32.7	15.8	28.5	13.8	27.6	27.9	28.6	27.6
30a-5p	30.9	27.4	23.7	22.9	22.7	25.5	23.5	22.7

## Data Availability

The original contributions presented in the study are included in the article; further inquiries can be directed to the corresponding author.
